# Editorial: Global excellence in rheumatology: Europe

**DOI:** 10.3389/fmed.2023.1242449

**Published:** 2023-06-29

**Authors:** Alessia Alunno, Chris Wincup, Meghna Jani, Dario Roccatello, Javier Rodríguez-Carrio

**Affiliations:** ^1^University of L'Aquila, MeSVA Department, Internal Medicine and Nephrology Division, ASL 1 Avezzano-Sulmona-L'Aquila, L'Aquila, Italy; ^2^King's College Hospital NHS Trust, London, United Kingdom; ^3^Department of Rheumatology, University College London, London, United Kingdom; ^4^Centre for Epidemiology Versus Arthritis, Centre for Musculoskeletal Research, The University of Manchester, Manchester, United Kingdom; ^5^University Center of Excellence on Nephrologic, Rheumatologic and Rare Diseases (ERK-Net, ERN-Reconnect and RITA-ERN Member), Nephrology and Dialysis Unit and Center of Immuno-Rheumatology and Rare Diseases (CMID), Coordinating Center of the Interregional Network for Rare Diseases of Piedmont and Aosta Valley (North-West Italy), Department of Clinical and Biological Sciences, University of Turin and San Giovanni Bosco Hub Hospital, ASL Città di Torino, Turin, Italy; ^6^Area of Immunology, Department of Functional Biology, Faculty of Medicine, University of Oviedo, Oviedo, Spain; ^7^Instituto de Investigación Sanitaria del Principado de Asturias (ISPA), Oviedo, Spain

**Keywords:** rheumatology, arthritis, machine learning, arthritis (including rheumatoid arthritis), lupus

Rheumatology is a rapidly evolving specialty where major breakthroughs have been produced in recent years, from a paradigm change in clinical management of rheumatic and musculoskeletal diseases (RMDs) to the discovery of novel drugs and biological agents to manipulate the immune response in the context of autoimmunity and autoinflammation. European rheumatology is to be commended for its contribution to the global excellence in rheumatology worldwide, including the development of evidence-based consensus recommendations for clinical management (1, 2), the development of robust methodologies to combine efforts from all stakeholders (rheumatologists, researchers, health professionals in rheumatology and people with RMDs) (3), the establishment of international collaborations across European countries using registries and databases (4–6), and the harmonization of training programs (7). Several of these achievements can be largely connected to the leadership of a common entity, the European Alliance of Rheumatology Associations (EULAR), who has served as an umbrella for clinical and research affairs in this setting. The present Research Topic provides a summary of the European excellence in rheumatology, covering the whole range from clinical science to basic and translational research, using a number of different approaches.

The need for novel targets in RMDs is an area of constant research, with the aims not only of establishing personalized medicine approaches but also to expand and refine the therapeutic armamentarium. This point is addressed in the present Research Topic in a series of review articles focused on two of the most prevalent immune-mediated RMDs: systemic lupus erythematosus (SLE) and rheumatoid arthritis (RA). Parodis et al. comprehensively summarized the potential of B-cell altering therapeutics, including B-cells, B-cell related cytokines, and plasma cells, as well as combination strategies. A special emphasis is made in the promising role of B-cells as biomarkers to guide such strategies, with the aim of maximizing clinical benefit, although the exact readouts are yet to be identified. In the case of RA, Gremese et al. advocated for the therapeutic potential of IL-8- and IL-17-targeted approaches based on the pathogenic role of these two molecules. In fact, this may explain at least in part the inadequate response of a proportion of RA patients to biological therapies with other mechanisms of action. A series of elegant experiments in animal and *in vitro* models as well as evidence from synovial studies reinforced this rationale, and candidate approaches, such as bi-specific antibodies or JAK inhibitors are discussed.

Basic and translational science has been also instrumental in expanding both the knowledge on RMDs pathogenesis and the therapeutic armamentarium. Barker et al. have reported a significant dysregulation of the mTOR pathway at synovial tissue level, also related to alterations in the Hippo-YAP pathway, leading to immunometabolism traits. The inhibitory effect of rapamycin may pave the ground for novel therapeutic strategies focused on these pathways.

The present Research Topic also showcases different examples of excellence in clinical science. In benefit of solid, evidence-based and good practices in research, the registration of study protocols (beyond industry-sponsored clinical trials) has become more frequent in recent years, and it is usually mandatory for certain institution and funding agencies. Following this step, Rademacher et al. have presented their protocol to evaluate the feasibility of rheopheresis for Raynaud's syndrome and digital ulcers in systemic sclerosis, also including a number of secondary endpoints (from imaging to patient-reported outcomes). The results of this apheresis approach are much awaited, since current evidence relies on retrospective studies or case reports, thus limiting clinical application. Another clinical study has shed new light on a highly relevant, although usually less attended, topic in RMDs care: the work participation. Dejaco et al. demonstrated that treatment with golimumab, a biological disease-modifying antirheumatic drug that blocks TNF, led to a significant improvement in work productivity and activity in real-world patients with RA, psoriatic arthritis and axial spondyloarthritis, with no major differences across the three patient groups. Although limited in follow-up (24 months), these results warrant further research into this area, with the ultimate goal of reducing work impairment in people with RMDs. Whether work participation outcomes should be considered to guide treatment recommendations need to be considered in future guidelines.

Finally, it must be recognized how much artificial intelligence and machine learning techniques are shaping our world and the way we interact with our environment, either in professional or personal domains (8). The paper by Knitza et al. introduces how machine learning approaches can be used to improve a rheumatology referral system, by including laboratory parameters and enabling individual feature adaptations. In a system of increasing clinical pressure and overloaded staff, machine learning approaches show promise to improve early referrals, a major unmet need in the clinical care of RMDs. Additionally, it may also have several benefits in the implementation of telemedicine in rheumatology, particularly in the post-COVID-19 era. However, trials addressing cost-effectiveness, clinical validation and cross-cultural adaptations are needed.

In summary, even in a challenging momentum for health systems, European rheumatology continues to show its hallmarks as a pioneer specialty. Multi-level approaches, high standards and solid methodological procedures to solve complex questions and cover the whole disease process are the pillars of the excellence of European rheumatology, with the ultimate goals of improving clinical care and patients' lives.

## Author contributions

JR-C edited the final version of the manuscript. All authors drafted, revised the manuscript, and approved the final version of the manuscript.
